# Efficient CO_2_ Capture Using Nitrogen-Enriched Microporous Carbon Derived from Polybenzoxazine in a Single-Step Process for Environmental Sustainability

**DOI:** 10.3390/polym17030343

**Published:** 2025-01-26

**Authors:** Thirukumaran Periyasamy, Shakila Parveen Asrafali, Jaewoong Lee

**Affiliations:** Department of Fiber System Engineering, Yeungnam University, Gyeongsan 38541, Republic of Korea; thiru.kumaran999@gmail.com (T.P.); shakilaparveen@yu.ac.kr (S.P.A.)

**Keywords:** polybenzoxazine, nitrogen-containing carbon, mesoporous, CO_2_ capture

## Abstract

In this research, we successfully synthesized nitrogen-enriched microporous carbon through a meticulous process involving two different activation procedures. Initially, polybenzoxazine was carbonized at 800 °C to create a precursor material, which was then activated with two different activating agents (KOH and KMnO_4_) at the same temperature. This activation significantly enhanced the material’s porosity, increasing its specific surface area from 335 m^2^/g (KOH activated) to 943 m^2^/g (KMnO_4_ activated). XPS analysis confirmed the presence of nitrogen functionalities, including secondary-N, oxide-N, pyridone-N, and pyridine-N, which are critical for CO_2_ adsorption. Adsorption tests demonstrated a high CO_2_ uptake of 3.8 mmol/g at 25 °C and 1 bar, driven by a combination of physisorption (physical interaction with the surface area) and chemisorption (chemical interaction with nitrogen sites). This high adsorption capacity can be attributed to the carbon’s substantial surface area, significant micropore volume, and the interconnected network of pores, which together provide structural stability and facilitate the diffusion of CO_2_ molecules. These findings suggest that this nitrogen-enriched microporous carbon, derived from polybenzoxazine, holds significant promise as a highly efficient material for applications in CO_2_ capture and storage.

## 1. Introduction

In the past two centuries, atmospheric greenhouse gas concentrations have surged dramatically. The concentration of carbon dioxide (CO_2_) has surged significantly, rising from around 280 parts per million (ppm) in the preindustrial era to over 420 ppm by 2023 [1,2,3]. This steep increase aligns with a global temperature rise of approximately 1.5 °C, highlighting a strong link between greenhouse gas accumulation and climate change. Studies overwhelmingly confirm that this warming trend is largely driven by human actions, with fossil fuel combustion playing a major role in escalating CO_2_ emissions [4,5,6,7,8]. The data point to an urgent need for mitigating human impact to limit further temperature increases and associated environmental consequences. During the economic slowdown from 2020 to 2022, CO_2_ emissions temporarily dropped, highlighting the impact of reduced industrial activity. However, such crises cannot be seen as a sustainable path to address climate change. Instead, more proactive and long-term solutions, like carbon capture and sequestration (CCS), should be adopted as practical approaches to curb CO_2_ emissions. CCS technology focuses on capturing CO_2_ emissions at their primary sources, such as power plants, before the gases reach the atmosphere. Once captured, the CO_2_ is stored in a way that prevents it from re-entering the atmosphere, typically in geological formations deep underground. By preventing CO_2_ from contributing to further warming, CCS presents a viable tool in the global effort to mitigate climate change while allowing for gradual transitions in energy systems [9,10,11,12,13].

To date, three primary technologies are employed for CO_2_ capture from industrial emissions: liquid scrubbing solutions, solid sorbents, and membrane filtration. Among these, solid sorbents offer several benefits, particularly for transport, potential recovery, or permanent sequestration, which includes metal–organic frameworks (MOFs), hyper-crosslinked polymers, conjugated microporous polymers, intrinsically microporous polymers, porous aromatic structures, microporous organic networks, porous polymer matrices, and benzimidazole-linked networks. However, some sorbents, such as MOFs, are highly sensitive to moisture, which limits their performance in humid conditions. Therefore, developing MOF with water resistant properties is challenging in CO_2_ capture. Additionally, the use of expensive and toxic solvents during MOF synthesis increases their production and application cost in large scale and creates environmental hazards. A significant challenge with many solid sorbents is their powdered form, which requires repackaging into structures that allow gases to flow freely through the material while maintaining adequate contact time for CO_2_ adsorption [14,15,16,17,18]. A promising approach for effective CO_2_ capture with solid sorbents involves merging catalytic conversion capabilities into a single material that features a hierarchical porous design. Aerogels, known for their multiscale nano-porous structures, may fulfill these requirements by enabling nearly unobstructed gas diffusion while providing ample surface area for CO_2_ uptake, making them a potentially ideal candidate for advanced CO_2_ capture solutions [19,20,21,22].

Despite its effectiveness, this method faces several challenges, including high energy demands, issues with equipment corrosion, significant operational costs, and potential environmental concerns, which restrict its widespread adoption. To address these limitations, adsorption using porous materials has emerged as a promising carbon capture and storage (CCS) technology. Porous carbon has recently emerged as a highly effective adsorbent for carbon dioxide (CO_2_) owing to its remarkable properties, including significant porosity, an extensive specific surface area, outstanding thermal stability, and lightweight nature. Moreover, the capture efficiency of CO_2_ can be further improved by modifying its surface with targeted atoms, ions, and molecules. However, the use of high temperature activation (between 800–1000 °C) for the preparation of porous carbons results in the release of CO_2_ into the atmosphere, leading to environmental issues. This could be mitigated through several methods involving greener synthesis, optimization of the activation processes–reducing the activation temperature while maintaining the porosity by using specific catalysts—and activation under controlled atmosphere using inert gases like N_2_ or Argon, which can prevent the oxidative release of CO_2_. Numerous studies have focused on creating nitrogen-functionalized porous materials for this purpose. Nitrogen-doped carbon materials (N-doped carbon) enhance CO_2_ capture due to their tailored surface chemistry, improved adsorption capabilities, and environmental stability. These materials leverage nitrogen’s ability to introduce polar functional groups, enhancing interactions with CO_2_ molecules. Nitrogen functional groups, such as pyridinic, pyrrolic, and quaternary nitrogen, with their basic chemical properties, create strong binding sites for CO_2_ through acid-base interactions, thus promoting efficient CO_2_ capture. N-doping often enhances material porosity by increasing the accessible surface area for CO_2_ capture. Additionally, N-doped carbons demonstrate high thermal and chemical stability, making them ideal for repeated CO_2_ adsorption–desorption cycles. This advancement in material modification holds significant potential for improving CCS technologies [23,24,25,26,27].

Nitrogen-enriched porous carbon can be synthesized using two primary techniques: post-synthetic amine modification and in situ synthesis. The post-synthetic approach generally exhibits lower efficiency in capturing carbon dioxide (CO_2_). This limitation arises because the method often leads to pore blockage and the agglomeration of nitrogen-doping agents, which consequently diminishes pore size within the carbon structure. Conversely, the in-situ synthesis method is favored due to its numerous advantages. It simplifies the overall preparation process, reduces the number of required steps, allows for greater control over the pore architecture, and facilitates a uniform distribution of nitrogen-active sites throughout the carbon matrix [28].

An example of the effectiveness of the in-situ method is demonstrated by Tseng et al. (2015), who successfully synthesized nitrogen-enriched porous carbon from melamine-modified phenol–formaldehyde resins. Their work showed notable CO_2_ capture performance, although it was limited to tests conducted at ambient pressure (1 bar). To expand the utility of nitrogen-enriched porous carbon for CO_2_ capture, future research should aim to optimize these materials by designing them with well-defined structures that enhance performance across a wider range of pressures. This will not only improve the effectiveness of CO_2_ capture but also contribute to more sustainable carbon management strategies in various applications. Ultimately, advancing the synthesis and optimization of nitrogen-rich porous carbons will play a crucial role in addressing global challenges related to carbon emissions and climate change [29,30,31].

In this study, a polymer precursor of polybenzoxazine (PBz) was synthesized using melamine, stearylamine, 2-tert-butylphenol, and formaldehyde. This paper presents a novel approach for synthesizing nitrogen-rich polymers using melamine, which serves both as a nitrogen source and a catalyst in the process. Modified from our previous works [32,33], this synthesis strategy incorporates many benzoxazine monomers in the branched structure, which in turn, results in the inclusion of several nitrogen atoms in the carbon framework. Through carbonization under a nitrogen atmosphere, these polymers are transformed into porous carbons with exceptionally high nitrogen content. Melamine contributes pyridinic and pyrrolic nitrogen species that are chemically integrated into the polymer and carbon frameworks. The resulting nitrogen-doped porous carbons are highly effective as CO_2_ adsorbents. Comprehensive characterizations of material synthesis, pore size determination, and CO_2_ adsorption studies are detailed in this study.

## 2. Methods

### 2.1. Synthesis of Multifunctional Benzoxazine from Melamine and Stearylamine (MSt-Bzo)

**Scheme 1:**
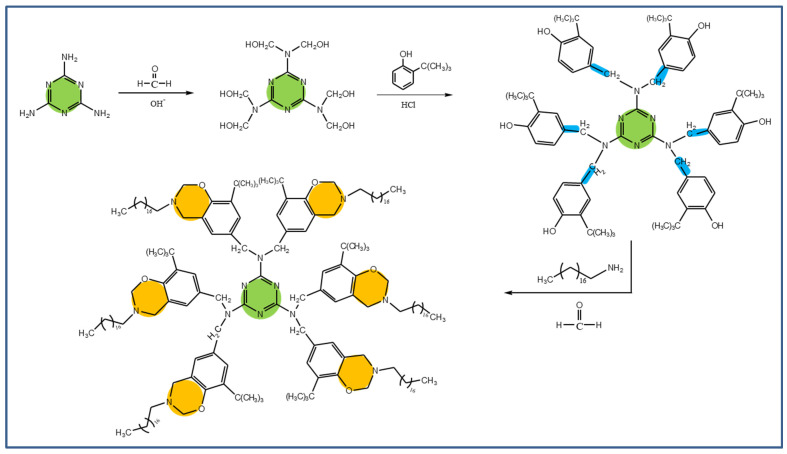
Synthesis of benzoxazine monomer, MSt-Bzo.

To synthesize a multifunctional benzoxazine, hexamethylol melamine was prepared through the reaction of melamine with formaldehyde. In this step, 1 mole of melamine was dissolved in 6 moles of aqueous formaldehyde solution, maintaining the pH at around 9–10 using a mild base such as sodium carbonate. The reaction should proceed under controlled temperatures, typically between 40–50 °C, for 12 h to ensure the complete formation of hexamethylol melamine. In the next step, the synthesized hexamethylol melamine was reacted with 2-(tert-butyl) phenol in presence of HCl as a catalyst. 2-(tert-butyl) phenol was added in smaller portions to the solution containing hexamethylol melamine and HCl, keeping the reaction temperature between 80–90 °C to facilitate the condensation reaction. This step results in the formation of a phenolic intermediate, which incorporates tert-butyl-substituted phenolic groups into the structure. The phenolic compound formed is crucial for subsequent benzoxazine ring formation. The next phase involves reacting the phenolic intermediate with additional formaldehyde and stearylamine. In this step, the phenolic intermediate was reacted with stearylamine and excess of formaldehyde, under reflux conditions, maintaining a temperature of 120 °C for 5 h to promote the formation of benzoxazine rings. The mixture was left to cool down gradually until it reached room temperature. Afterward, it was precipitated by adding it to a 1N NaOH solution. The formed precipitate was collected and washed multiple times with distilled water to ensure the removal of any unreactants. Subsequently, it was filtered and then dried under vacuum conditions at 60 °C for a duration of 12 h, ultimately producing the MSt-Bzo monomer (Scheme 1). Throughout this process, the combination of formaldehyde with phenol and stearylamine leads to the development of a multifunctional benzoxazine resin.

### 2.2. Fabrication of Nitrogen-Doped Porous Carbon Materials

**Scheme 2:**
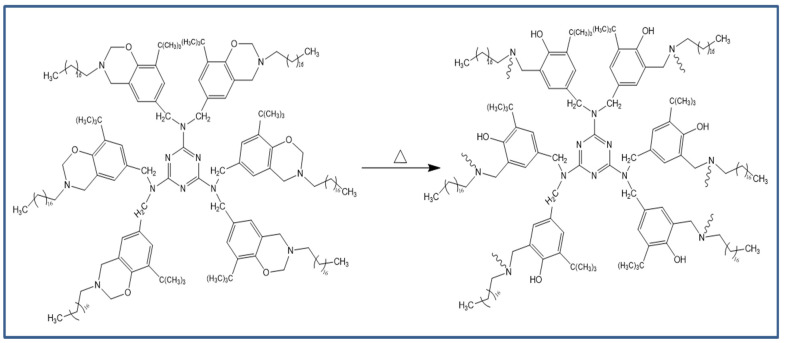
Polymerization mechanism of benzoxazine monomer.

The synthesized benzoxazine monomer was gradually heated in an oven in a stepwise manner following the heating schedule of 100, 150, 200, and 250 °C, with a holding time of 1 h at each temperature to ensure complete curing process. This resulted in the formation of polybenzoxazine (PBz) in Scheme 2, which was then carbonized. Carbonization of the sample was performed under an argon atmosphere. The sample was gradually heated to 600 °C at a rate of 5 °C per minute and maintained at this temperature for 5 h. The resulting carbonized material then underwent activation using two separate methods. In the first activation method, the carbonized sample was combined with KOH in a weight ratio of 2:1 (KOH: sample) and finely ground to ensure thorough mixing. The prepared mixture was heated to 800 °C (the activation temperature was fixed to 800 °C based on our previous studies) [32,33] under a continuous flow of argon gas, using a heating rate of 5 °C per minute, and held at that temperature for 1 h. Once the heating process was complete, the activated material was allowed to cool. It was then washed multiple times using 1 M HCl and deionized water until neutral pH was obtained. Finally, the material was dried at 110 °C for 12 h to remove residual moisture. For the second activation method, KMnO_4_ was used instead of KOH. The choice of KMnO_4_ is due to the following reasons: KMnO_4_ is a strong oxidizing agent and it is expected to introduce oxygen-containing functional groups like -COOH and -OH on the carbon surface. These groups increase hydrophilicity and binding sites for adsorption. Moreover, KMnO_4_ tends to etch carbon structures at a controlled rate, favoring the development of micropores and preserving structural stability. The activation steps were otherwise identical to the first method. The two final activated samples were designated as PBZC-KOH and PBZC-KMnO_4_, corresponding to the use of KOH and KMnO_4_, respectively. Yield of the carbon materials: 37–42%.

## 3. Results and Discussion

### 3.1. Structural Characterization of Benzoxazine Monomer (MSt-Bzo)

**Figure 1:**
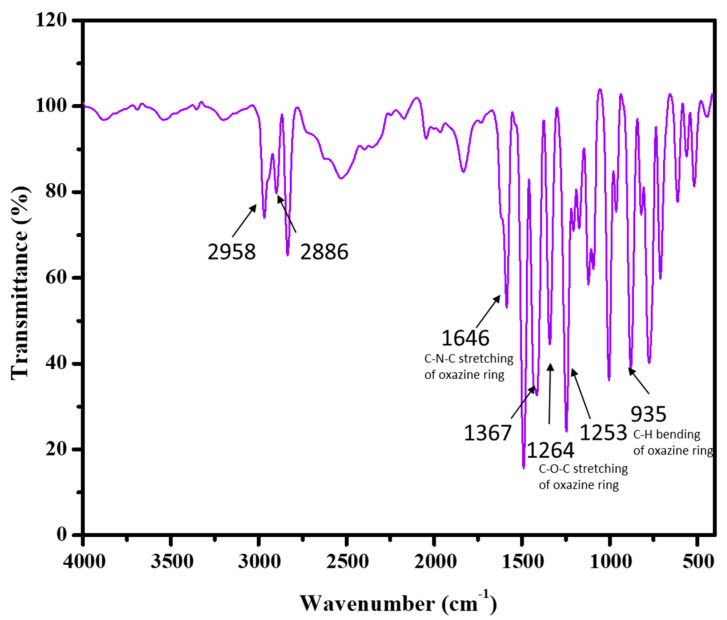
FT-IR spectrum of benzoxazine monomer, MSt-Bzo.

The successful synthesis of the MSt-Bzo benzoxazine monomer was confirmed using an array of analytical techniques, primarily FT-IR and ^1^H-NMR spectroscopy, which provided comprehensive structural insights. In the FT-IR spectrum (Figure 1), several distinct absorption bands reveal important details about the functional groups present in the compound. Notably, peaks observed at 1264 and 1253 cm^−1^ correspond to C-O-C stretching vibrations, confirming the establishment of the benzoxazine ring structure. A peak at 935 cm^−1^ further supports this by indicating the presence of the characteristic C-H bending mode associated with the benzoxazine group. An absorption band at 1646 cm^−1^ signals the existence of C-N-C of benzoxazine group. The peaks at 2958 and 2886 cm^−1^ correspond to the asymmetric and symmetric C-H stretching of the benzene ring and the stearylamine group, respectively, highlighting both aromatic and aliphatic regions within the molecule. Additionally, the band at 1367 cm^−1^ is attributed to the tetra-substituted benzene ring, further supporting the complexity of the compound’s structure [34,35].

**Figure 2:**
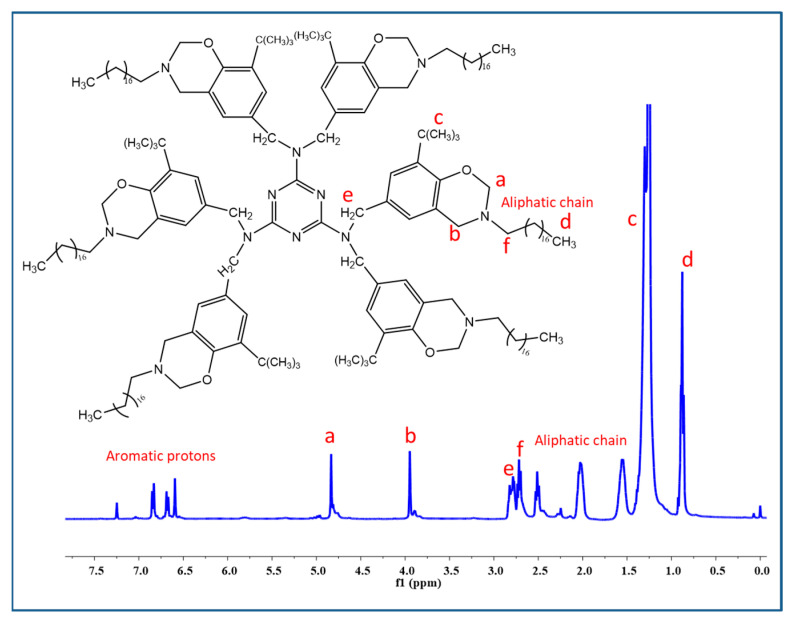
^1^H-NMR spectrum of benzoxazine monomer, MSt-Bzo.

The ^1^H-NMR and ^13^C-NMR spectrum (Figure 2 and Figure S1) offers further confirmation and additional structural details. Signals observed at 3.9 and 4.8 ppm correspond to protons in the oxazine ring, confirming the presence of this critical functional group. The region between 0.5 and 3.0 ppm shows a series of peaks that are associated with the aliphatic chain of the stearylamine moiety. The aromatic protons within the benzene rings produce multiplets between 6.5 and 7.0 ppm, adding further evidence to the molecular complexity and confirming the presence of aromatic structures. These spectral features, collectively, provide a detailed understanding of the structural composition of the synthesized MSt-Bzo precursor [36,37].

### 3.2. Structural Characterization of the Carbon Materials

**Figure 3:**
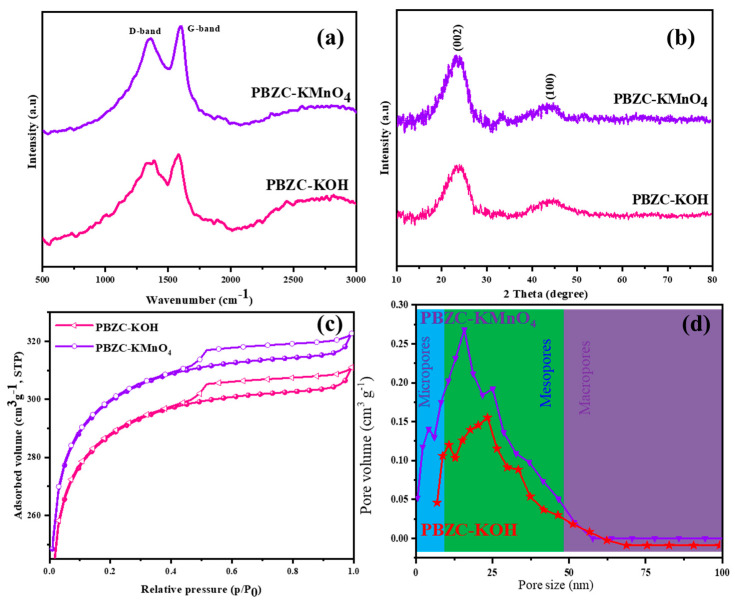
(**a**) Raman spectra; (**b**) XRD patterns; (**c**) N_2_ adsorption-desorption isotherms; and (**d**) pore size distribution curves of porous carbon materials.

The synthesis of heteroatom-containing carbons derived from polybenzoxazine was successfully achieved through a process involving a Mannich reaction, followed by carbonization and chemical activation. This multi-step method led to the creation of porous carbons with high surface areas and unique structural properties. By activating these materials with KOH and KMnO_4_, the resulting carbons exhibit distinct graphitic characteristics, as evidenced by means of Raman spectroscopy and X-ray diffraction (XRD) analyses. Raman spectroscopy in Figure 3a revealed critical details about the structure of the KOH-activated (PBZC-KOH) and KMnO_4_-activated (PBZC-KMnO_4_) carbons. Both samples exhibited two distinct peaks at approximately 1356 and 1585 cm^−1^, corresponding to the characteristic ‘D’ and ‘G’ bands, respectively. The ‘D’ band is associated with defects or disorder in the carbon structure, highlighting areas where the lattice is disrupted. On the other hand, the ‘G’ band is indicative of graphitic regions, reflecting the presence of well-ordered carbon domains. In both of the samples, the pronounced intensity of the D band suggests a significant concentration of structural imperfections [38]. This is likely due to the incorporation of nitrogen and oxygen atoms into the carbon framework, which disrupts its regularity and introduces additional defects. These structural defects due to hetero-atom doping, expose active sites on the carbon surface where CO_2_ molecules can form chemical bonds, facilitating chemisorption over physisorption. Additionally, these defective sites anchor heteroatoms strongly, leading to specific interactions with CO_2_ through acid-base mechanisms. Moreover, the presence of structural defects alters the electronic structure of the carbon material, increasing the density of localized electronic states that enhance interactions with CO_2_. The ratio of the D to G band intensities (I_D_/I_G_) serves as a measure of graphitization, where lower values indicate a more graphitic (ordered) structure. For PBZC-KOH and PBZC-KMnO_4_, the I_D_/I_G_ ratios were calculated as 0.88 and 0.94, respectively. The slightly higher I_D_/I_G_ ratio of PBZC-KMnO_4_ suggests that activation by KMnO_4_ at higher temperatures may promote a modest increase in structural defects by removing some nitrogen and oxygen atoms, preserving the materials’ original structure.

The X-ray diffraction (XRD) patterns (Figure 3b) provided additional confirmation about the existence of graphitic structures in both samples. Upon performing wide-angle XRD analysis, two distinct broad diffraction peaks were observed at approximately 23.5° and 45°. These peaks are characteristic of the (002) and (100) planes that are typically associated with graphitic carbon. Notably, the intensity of the (100) peak was somewhat diminished, suggesting enhanced degree of graphitization within the samples. This reduction in peak intensity points to an increase in the orderliness and crystalline structure of the graphite present. This effect is likely due to the high-temperature activation process, which facilitates the formation of graphitic domains. The interlayer spacing (d-spacing) was calculated using Bragg’s equation and found to be approximately 0.378 nm for both materials, which is consistent with the dimensions typically observed in graphitic carbon [39,40]. This characteristic spacing is favorable for use in supercapacitors, as it enhances the material’s capacitance and electrical conductivity, making it suitable for energy storage applications.

### 3.3. BET Analysis of the Carbon Materials

Nitrogen adsorption–desorption isotherms were employed to analyze the pore structures of the activated carbons. As seen in Figure 3c, both PBZC-KOH and PBZC-KMnO_4_ exhibited similar hysteresis loops, characteristic of mesoporous materials. At relative pressures below 0.1, a sharp increase in nitrogen adsorption indicated the presence of micropores, while a continued rise in adsorption at relative pressures up to 0.9 suggested mesopore filling. Beyond P/P_0_ > 0.9, a pronounced increase in adsorption signified the presence of macropores. The surface areas calculated via the Brunauer–Emmett–Teller (BET) method demonstrated that PBZC-KOH and PBZC-KMnO_4_ possessed surface areas of 335 and 943 m^2^/g, respectively, highlighting the impact of activation type on porosity and surface area. The use of KOH and KMnO_4_ as activation agents had a notable influence on the pore sizes and distribution within the materials. For instance, the activation with KMnO_4_ at 800 °C resulted in the formation of micropores within the PBZC-KMnO_4_ structure, with sizes ranging from 1 to 10 nm (see Figure 3d). These extreme activation conditions caused partial etching of the carbon structure, expanding the mesopores while retaining a high degree of microporosity. Simultaneously, balancing microporosity and mesoporosity is essential for effective gas diffusion and adsorption. Micropores provide a high surface area with abundant adsorption sites, while mesopores act as diffusion channels, ensuring CO_2_ molecules reach inner regions without blockage and minimizing resistance. As KMnO_4_ is a strong oxidizing agent, prolonged exposure at high temperatures can result in over-oxidation, reducing carbon yield and introducing undesirable surface functionalities that may reduce stability of the materials. However, in this work, activation process was performed under inert conditions to reduce the risk of over-oxidation and material collapse. Even under these rigorous activation processes, the incorporation of nitrogen and oxygen atoms within the carbon matrix was preserved, enhancing the thermal and chemical stability of the materials. This stability is advantageous for applications requiring resilient materials, such as high-performance supercapacitors [41,42,43].

Thermal treatment of the polybenzoxazine precursor yielded a high char content, approximately 50%, positioning it as an ideal source material for developing heteroatom-enriched carbons. The embedded nitrogen and oxygen atoms add functional versatility and stability to the carbon structure. This heteroatom incorporation is particularly beneficial for supercapacitor applications, where high surface area, conductivity, and chemical stability are essential for optimal energy storage performance. Additionally, the unique pore structure generated by this method enhances ion transport within the material, which can further boost the capacitance and charge–discharge capabilities of the supercapacitors. Overall, the synthesis process of PBZC-KOH and PBZC-KMnO_4_ highlights the effectiveness of polybenzoxazine-derived carbons in achieving high surface area, favorable graphitic properties, and robust structural stability. The variations in activation conditions by KOH or KMnO_4_ demonstrate the ability to tune the porosity and graphitization level, allowing these materials to be tailored for advanced CO_2_ capture applications.

### 3.4. XPS Analysis of the Carbon Materials

**Figure 4:**
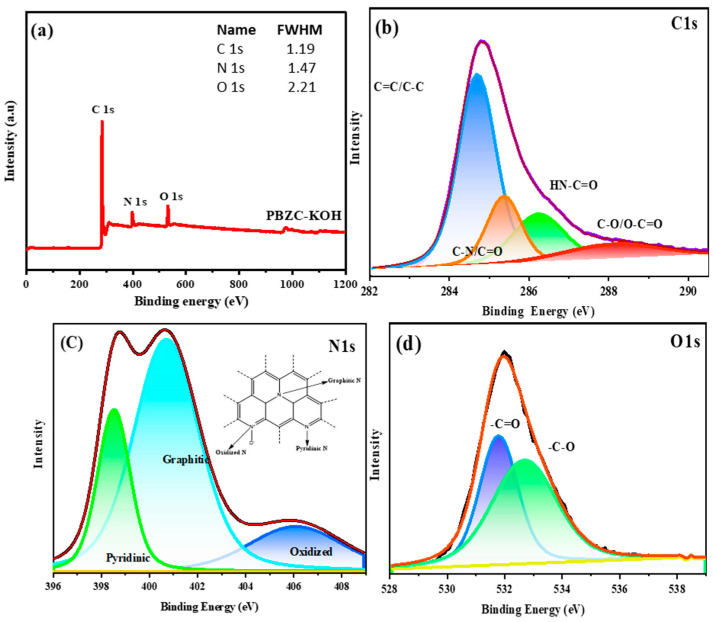
XPS analysis showing the (**a**) survey spectrum; and (**b**–**d**) deconvoluted spectra of PBZC-KOH.

**Figure 5:**
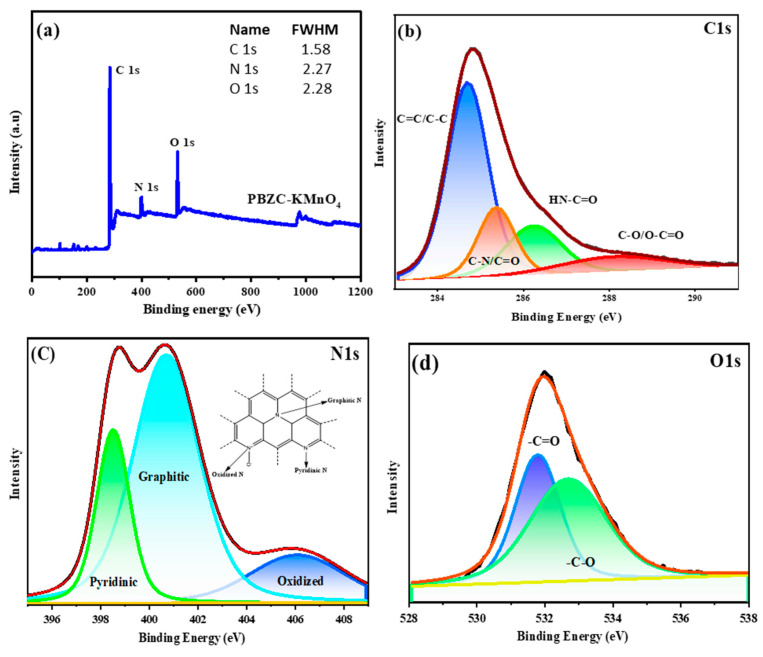
XPS analysis showing the (**a**) survey spectrum; and (**b**–**d**) deconvoluted spectra of PBZC-KMnO_4_.

The X-ray Photoelectron Spectroscopy (XPS) analysis presented in Figure 4 and Figure 5 reveals the detailed chemical state and bonding environment of carbon, nitrogen, and oxygen atoms in the porous carbon samples, PBZC-KOH and PBZC-KMnO_4_. The wide-scan XPS spectra for both samples show prominent peaks corresponding to C 1s, N 1s, and O 1s, emphasizing the presence of these elements derived from the benzoxazine monomer precursor, which is rich in nitrogen and contains oxygen. The spectra confirm that the carbon materials are of high purity, as there are no extraneous elements detected. The primary photoelectron peaks are observed at binding energies of approximately 286.1 eV for C 1s, 398.9 eV for N 1s, and 532 eV for O 1s, indicative of characteristic carbon, nitrogen, and oxygen bonds. The high-resolution C 1s spectra (Figure 4b and Figure 5b) are deconvoluted into four distinct peaks: The peak at 284.3 eV corresponds to graphitic sp^2^ carbon (C=C/C-C), while the peak at 285.2 eV is attributed to carbon atoms bonded to nitrogen in C-N configurations. The peak at 285.8 eV indicates carbon atoms bonded to both oxygen and nitrogen in groups such as HN-C=O, and the peak at 288.3 eV signifies the presence of oxygenated groups like O-C=O/C-O and hydroxylated carbon [44,45,46,47,48].

The N 1s spectra (Figure 4c and Figure 5c) demonstrate multiple types of nitrogen species in the carbon matrix, including pyridinic nitrogen at 398.3 eV, graphitic nitrogen at 400.2 eV, and oxidized nitrogen species at around 404.7 eV. These nitrogen functionalities are crucial for enhancing the electrochemical properties of the porous carbon. Pyridinic nitrogen, bonded to two carbon atoms at the edges of the carbon lattice, provides strong Lewis base sites that interact with the acidic CO_2_ molecule via electron donation and enhance chemisorption, improving binding strength and selectivity. Graphitic nitrogen, integrated into the graphitic lattice, alters the electronic properties of the carbon material and improves the interaction strength with CO_2_ through polarization effects. This type of nitrogen enhances physisorption by creating an electron-rich surface and contributes to long-term stability of the material under repeated adsorption-desorption cycles. The high polarity of the oxidized nitrogen species creates strong electrostatic attractions with CO_2_. Synergistic effects produced when combining pyridinic, graphitic, and oxidized nitrogen help to improve overall CO_2_ uptake. Pyridinic nitrogen is particularly important for providing higher capacitances through redox reactions in acidic and alkaline solutions, contributing to the pseudo-capacitive behavior of the material. The graphitic nitrogen species, which enhance electron conductivity, play a vital role in improving the overall electrical performance of the carbon material [45,47,49]. Furthermore, the degree of graphitization, evident from the XPS analysis, significantly affects the conductivity of PBZC.

The O 1s spectra (Figure 4d and Figure 5d) are deconvoluted into two peaks at 531.5 and 533.2 eV, corresponding to quinones (C=O) and ether or hydroxyl groups (C-O-C), respectively. These oxygen functional groups, while not directly contributing to electrochemical activity, can facilitate reversible redox reactions in an alkaline medium. Phenolic hydroxyls, for example, exhibit quasi-reversible pseudo-capacitance behavior through deprotonation [49,50,51,52,53,54]. The carbonyl and ether oxygen groups in the material enhance its capacity for CO_2_ capture by promoting effective interactions and adsorption processes. The interplay between nitrogen species, graphitization levels, and oxygen functionalities contributes to the material’s unique surface characteristics, facilitating efficient CO_2_ adsorption and retention. These properties make the porous carbon material highly suitable for CO_2_ capture applications, where both physical and chemical adsorption mechanisms are crucial.

### 3.5. Morphological Analysis of the Carbon Materials

**Figure 6:**
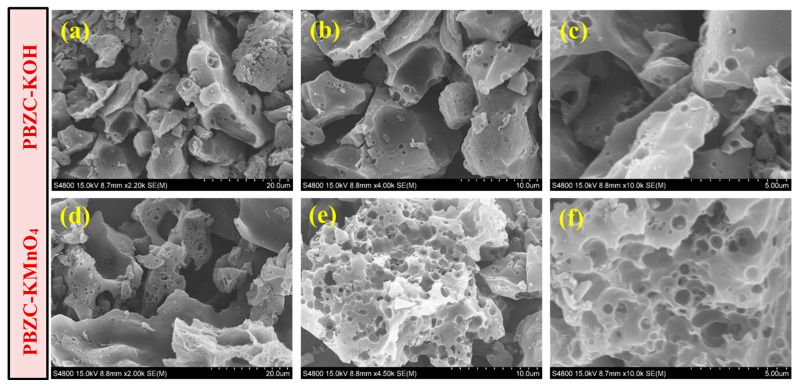
SEM images of (**a**–**c**) PBZC-KOH; and (**d**–**f**) PBZC-KMnO_4_ at different magnifications.

As illustrated in Figure 6, different activation procedures lead to variations in the surface morphology and pore distribution of activated carbon materials. The SEM images highlight how activation conditions impact the porosity and surface features of the resulting materials. For PBZC-KOH (Figure 6a–c), the images reveal numerous irregular micropores with a range of sizes, alongside only a few mesopores. This porosity pattern arises from the relatively low activation temperature of 800 °C, which results in limited pore development. The carbon structure retains many small, dense features with minimal large pores, emphasizing the microporous nature created under these conditions. In contrast, PBZC-KMnO_4_, subjected to activation at the same temperature (800 °C), exhibits a more complex pore architecture, as shown in Figure 6d–f. The SEM images depict a collapsed and fragmented carbon structure with a higher prevalence of voids. A mix of micro-, meso-, and macropores emerges, with pore sizes spanning from 1 to 100 nm. The activation with KMnO_4_ produces a greater number of macropores, leading to a structure characterized by thin pore walls and larger interconnected cavities. The data clearly demonstrate that optimizing the activation temperature and agent can tailor the pore structure and size distribution of these carbon materials. Specifically, higher temperatures and stronger oxidizing agents like KMnO_4_ promote the formation of macropores and interconnected networks, while lower temperatures favor micropore development. The choice of activation parameters thus plays a critical role in customizing the material’s porosity for specific applications, such as CO_2_ capture, energy storage, and so on.

**Figure 7:**
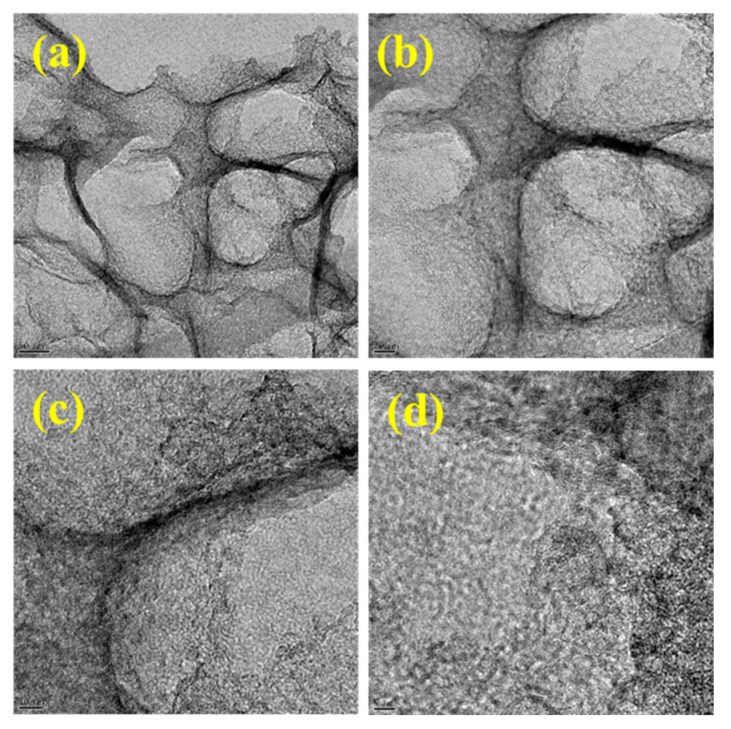
(**a**–**d**) TEM images of PBZC-KMnO4 at different magnifications.

**Figure 8:**
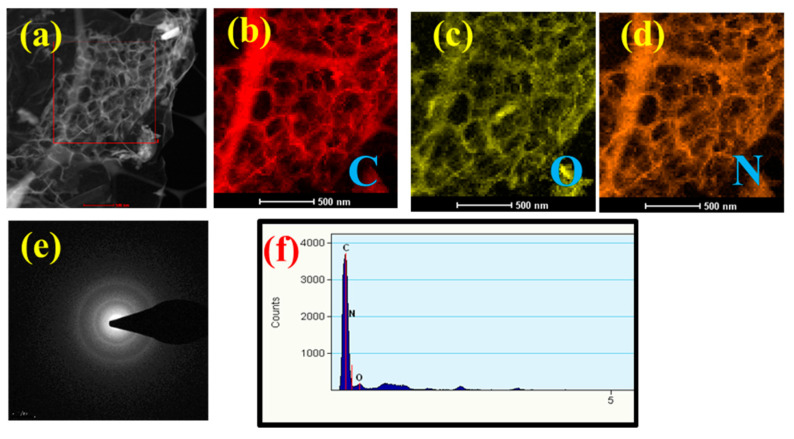
(**a**–**d**) Elemental mapping; (**e**) SAED pattern; and (**f**) EDX spectrum of PBZC-KMnO4.

The TEM images and elemental mapping in Figure 7 provide a comprehensive analysis of the microstructure and elemental distribution of PBZC-KMnO_4_. The high-resolution TEM images (Figure 7a–d) reveal the formation of well-dispersed carbon nanostructures. These images highlight the presence of dense and interconnected porous networks. The edges and surfaces of these carbon structures appear irregular, with varying thickness and degrees of crystallinity, indicating the structural transformation induced by activation with KMnO_4_. Figure 7a,b shows relatively larger carbonaceous aggregates, while Figure 7c,d illustrates regions with finer and more disordered textures. The porous architecture observed is characterized by interconnected networks that create efficient channels, beneficial for applications like gas adsorption and ion transport. The elemental mapping (Figure 8a–d) further clarifies the distribution of key elements within the PBZC-KMnO_4_ structure. The mapping images indicate that carbon (C), oxygen (O), and nitrogen (N) are uniformly dispersed throughout the material. The red color represents carbon, confirming its presence as the primary constituent. The oxygen (yellow) and nitrogen (orange) are distributed evenly, suggesting successful integration of heteroatoms, which can enhance the chemical functionality of the material and improve its adsorption properties. The selected area electron diffraction (SAED) pattern (Figure 8e) displays a characteristic ring structure, indicating the presence of both amorphous and graphitic domains within the PBZC-KMnO_4_ sample. This mixed phase confirms partial graphitization, which is crucial for balancing electrical conductivity and porosity. The EDX spectrum (Figure 8f) shows the quantitative analysis of elements, highlighting strong peaks for carbon, with smaller yet significant peaks for oxygen and nitrogen. These heteroatoms are essential for imparting improved surface reactivity, enhancing CO_2_ capture and electrochemical performance. The data collectively reveal that PBZC-KMnO_4_ features a tailored porous structure and a strategic distribution of elements, making it well-suited for advanced environmental and energy applications.

### 3.6. CO_2_ Adsorption Studies for the Carbon Materials

**Figure 9:**
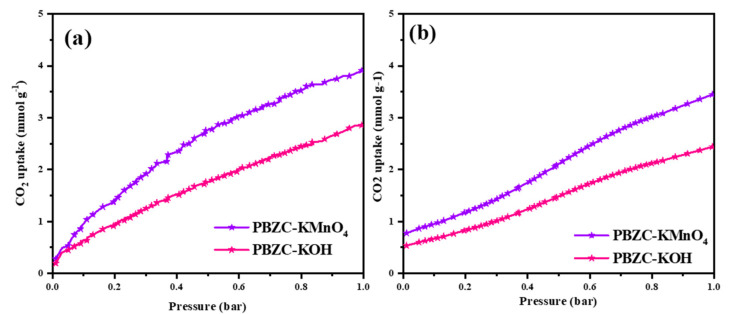
CO_2_ adsorption isotherms for the carbon materials measured at (**a**) 25 and (**b**) 0 °C.

CO_2_ adsorption/desorption isotherms were acquired by Tristar II 3020 gas sorption analyzer (micromeritics, Norcross, GA, USA) using CO_2_ as a carrier gas in the absolute pressure range between 10 and 850 Torr with a step change of 3–20 mmHg (or Torr) at 0 and 25 °C. Powder samples (about 80 mg) were loaded into a quartz sample tube and degassed by a sample degassing system (VacPrep 061 degasser, micromeritics, Norcross, GA, USA) at 150 °C under vacuum for 24 h before the sorption analysis. Then, the equilibrium weight of the powder sample was recorded and used in evaluating its CO_2_ capture performance with the amount of CO_2_ adsorbed at 850 Torr extracted from isotherms. The CO_2_ adsorption isotherms for two different activated carbons, PBZC-KOH and PBZC-KMnO_4_, tested at two temperatures: 25 and 0 °C are given in Figure 9a,b. The adsorption behavior of CO_2_ on these materials offers insight into their effectiveness for carbon capture applications, as CO_2_ uptake at different pressures and temperatures reflects their surface characteristics and pore structures. At 25 °C (Figure 9a), the CO_2_ adsorption capacity of both PBZC-KOH and PBZC-KMnO_4_ increases with rising pressure, indicating typical physisorption behavior. PBZC-KMnO_4_, activated with potassium permanganate, consistently shows a higher CO_2_ uptake than PBZC-KOH across the entire pressure range. The CO_2_ uptake reaches around 3.8 mmol/g for PBZC-KMnO_4_ and approximately 2.9 mmol/g for PBZC-KOH at 1 bar pressure. This higher CO_2_ capture capacity in PBZC-KMnO_4_ may be attributed to its larger surface area and higher microporosity, which provide more adsorption sites and improved accessibility for CO_2_ molecules [55,56,57,58]. The presence of heteroatoms, such as nitrogen and oxygen, likely enhances CO_2_ affinity due to their polar nature, facilitating stronger interactions with CO_2_ molecules.

When the temperature is lowered to 0 °C (Figure 9b), the CO_2_ adsorption capacity increases significantly for both materials. At lower temperatures, the kinetic energy of CO_2_ molecules decreases, favoring stronger physisorption interactions with the carbon surface. PBZC-KMnO_4_ again demonstrates a superior CO_2_ uptake, approaching 3.5 mmol/g, compared to PBZC-KOH, which captures slightly over 2.45 mmol/g at 1 bar. This enhanced performance at reduced temperatures highlights the potential of these materials for CO_2_ capture under various environmental conditions, particularly in applications where lower temperatures are feasible [59,60,61]. Overall, PBZC-KMnO_4_ outperforms PBZC-KOH in CO_2_ capture at both temperatures, likely due to its superior porosity, surface area, and the synergistic effects of heteroatom doping, which contribute to enhanced CO_2_ adsorption. This behavior suggests that KMnO_4_-activated polybenzoxazine-derived carbon (PBZC-KMnO_4_) is a promising candidate for efficient CO_2_ capture applications, especially in conditions where physisorption can be maximized (Table 1).

Even though the use of fine or ultrafine sorbent particles in CO_4_ capture offers advantages like high surface area and enhanced reactivity, their large-scale utilization poses several challenges related to material handling, stability, and efficiency. These fine particles tend to agglomerate due to high surface energy, which reduces the effective surface area and adsorption capacity. This could be avoided by using surfactants or surface functionalization to reduce particle–particle interactions. Ultrafine particles have poor flow properties, making their handling difficult during processes like fluidization or transport. Granulation or use of hybrid systems can improve flowability while maintaining reactivity. Fine particles are easily aerosolized, posing health risks to workers and environmental hazards if not contained. Implementing stringent containment measures, use of personal protective equipment, and developing safer forms of the material (like encapsulation) can overcome this issue. Regenerating fine sorbent particles can lead to material losses and reduced effectiveness. Using structured sorbents (honeycomb monoliths) and enhancing thermal or chemical stability aid in easier regeneration.

## 4. Conclusions

In this study, we developed nitrogen-enriched porous carbon using two activation agents, potassium hydroxide (KOH) and potassium permanganate (KMnO_4_), to investigate their effectiveness for CO_2_ capture applications. The initial carbonization of polybenzoxazine precursor at 800 °C led to a high-surface-area carbon framework. The subsequent activation revealed notable differences between the two methods. The KMnO_4_-activated carbon (PBZC-KMnO_4_) outperformed the KOH-activated carbon (PBZC-KOH), exhibiting a higher surface area of 943 m^2^/g compared to 335 m^2^/g for PBZC-KOH. This is attributed to the formation of a hierarchical pore structure comprising micro-, meso-, and macropores in PBZC-KMnO_4_, which enhances CO_2_ adsorption efficiency. The structural differences, confirmed by XRD and SEM analyses, showed that KMnO_4_ activation generates a diverse pore network and improves graphitization, leading to better CO_2_ uptake due to increased accessibility and diffusion channels within the carbon structure. CO_2_ adsorption tests at 25 °C indicated a maximum uptake of 3.8 mmol/g for PBZC-KMnO_4_, significantly higher than PBZC-KOH. This enhanced CO_2_ capture capacity is supported by the presence of nitrogen and oxygen functionalities, which strengthen CO_2_ interactions. In addition, the PBZC-KMnO_4_’s superior micro–meso–macro pore distribution and higher graphitization make it a promising candidate for CO_2_ capture. The study demonstrates that KMnO_4_ activation effectively tailors pore architecture and surface chemistry, optimizing the material for environmental applications in CO_2_ capture and storage. However, in large-scale applications, challenges including high production costs, limited recyclability, and safety issues associated with handling KMnO_4_ should be taken into concern.

## Figures and Tables

**Table 1:** Data showing the comparative analysis of CO_2_ uptake for different activated carbons.

Material	Nitrogen Content (%)	Nitrogen Species Distribution (Pyridinic, Pyrrolic, etc.)	BET Surface Area (m^2^/g)	CO_2_ Uptake Capacity (mmol/g)	Reference
PBZC-KMnO_4_	5.8	Pyridinic (45%), Graphitic (35%), Oxidized-N (20%)	943	3.8	This study
Epoxy nitrogen doped adsorbent (EN-450-2)	2.62	Pyrrolic N (61.95%) Pyridinic N (20.11%) Graphitic N (17.94%)	1920	3.14	[26]
Heteroatom containing porous carbon (HCPC-800)	6.26	-	984	3.6	[33]
Conjugated microporous polymer (CMP-1-COOH)	-	-	522	1.60	[46]
Polypyrrole (PPy/KOH)	10.1	Pyrrolic/Pyridonic N (84%) Pyridinic N (16%)	1700	3.9	[52]
Melamine-Phenol Resin Carbon	4.2	Pyridinic (40%), Pyrrolic (30%), Quaternary-N (30%)	800	2.9	[54]
MOF-derived Carbon	6.0	Pyridinic (50%), Quaternary-N (20%), Oxidized-N (30%)	1050	4.1	[56]
Activated Carbon (KOH)	3.5	Pyrrolic (50%), Oxidized-N (50%)	500	2.1	[61]

## Data Availability

The data presented in this study are available in the article.

## Supplementary Materials

The following supporting information can be downloaded at: https://www.mdpi.com/article/10.3390/polym17030343/s1, Details regarding the materials used, instruments used for characterizing the synthesized materials, ^13^C-NMR, DSC, and TGA are given in the supporting information. Figure S1: (a) ^13^C-NMR (b) DSC of MSt-Bzo and (c), TGA of poly (MSt-Bzo).
