# Supplementary material for: Efficient CO2 Capture Using Nitrogen-Enriched Microporous Carbon Derived from Polybenzoxazine in a Single-Step Process for Environmental Sustainability
Source: Polymers (Basel). 2025 Jan 26;17(3):343. doi: 10.3390/polym17030343 (PMC11820653; doi:10.3390/polym17030343)
Supplement: Supplementary file 1 [file polymers-17-00343-s001.zip › polymers-3442392-supplementary.pdf]

Efficient CO_2 Capture Using Nitrogen-Enriched Ultra-Microporous Carbon Derived from Polybenzoxazine in a Single-Step Process for Environmental Sustainability

Thirukumaran Periyasamy†, Shakila Parveen Asrafali†, and Jaewoong Lee*

Department of Fiber System Engineering, Yeungnam University, Republic of Korea.

Supplementary Information

Materials

Melamine, paraformaldehyde, stearylamine, and 2-(tert-butyl) phenol, were purchased from Sigma-Aldrich, St. Louis, MO, USA. Dimethyl sulfoxide (DMSO) and sodium hydroxide (NaOH) were provided by Duksan Chemicals Co., Ltd., Ansan-si, Republic of Korea. Potassium hydroxide (KOH) and potassium permanganate ($KMnO_4$) were obtained from Daejung Chemical Company, Ltd., Seoul, Republic of Korea. Hydrochloric acid (HCl, 35%) was sourced from Alfa Aesar. All chemicals were used as supplied, with no additional purification processes.

Characterization

To gain a comprehensive understanding of the sample, a variety of advanced analytical methods were utilized. Fourier transform infrared (FT-IR) spectroscopy was carried out using a Perkin Elmer MB3000 instrument (Hopkinton, MA, USA), spanning the wavenumber range of 400–4000 cm^{-1} with a resolution of 4 cm^{-1}, to detect and identify functional groups present in the material. Further structural characterization was achieved with nuclear magnetic resonance (NMR) spectroscopy. This analysis was conducted on an Agilent AVANCE NEO600 spectrometer (Oxford, UK) at a frequency of 600 MHz to investigate proton environments and other key structural features.

The microscopic morphology and elemental composition of the sample were analyzed using field emission scanning electron microscopy (FESEM) coupled with energy-dispersive X-ray spectroscopy (EDS). These measurements were taken on a Hitachi S-4800 microscope (Ibaraki, Japan) with an accelerating voltage set at 4 kV. Additionally, high-resolution transmission electron microscopy (HRTEM) was employed for detailed structural imaging. This was performed on a FEI-Tecnai TF-20 microscope operating at 120 kV to capture fine structural details.

Crystallographic information was obtained via X-ray diffraction (XRD) using a PANalytical X'Pert3 MRD diffractometer. The analysis utilized Cu Kα radiation (λ = 1.5406 Å) at 40 kV and 30 mA, with a scanning range of 10° to 80° in 2θ. Raman spectroscopy was also performed using a Horiba XploRA Micro-Raman system (Palaiseau, France) to analyze vibrational modes over the range of 500–3000 cm^{-1}. The wavelength of the laser used is 532 nm, as this directly influences the Raman signal and spectral features. It is also important to provide the laser power 10 mW, exposure time 30 seconds and the number of scans averaged 10 scans. Furthermore, the spectral resolution 1 cm^{-1} and the spectral range measured (500–3000 cm^{-1}). These parameters are necessary to ensure that the experimental setup can be replicated and the results accurately interpreted.

To evaluate surface area, pore size distribution, and pore volume, nitrogen adsorption–desorption isotherms were measured at −197 °C using a Micromeritics ASAP 2000 system. The samples were degassed at 120 °C, followed by an evacuation under argon at 140 °C for 8 hours. The Brunauer–Emmett–Teller (BET) and Barrett–Joyner–Halenda (BJH) models were applied to interpret the isotherm data.

Finally, X-ray photoelectron spectroscopy (XPS) was conducted to analyze the surface chemical composition and electronic states. This analysis utilized a Thermo Fisher Scientific K-Alpha spectrometer (Waltham, MA, USA). The radiation source used is Al Kα at 1486.6 eV along with the power settings 12 kV. Other parameters, such as the Gauss-Lorentz ratio 70:30, baseline correction method Shirley, and the fitting procedure for peak analysis are used for XPS spectra decomposition. Spectral deconvolution was performed using CasaXPS software. All analyses were supported by resources from the core research center at Yeungnam University.

CO_2 adsorption/desorption isotherms were acquired by Tristar II 3020 gas sorption analyzer (micromeritics, USA) using CO_2 as a carrier gas in the absolute pressure range between 10 and 850 Torr with a step change of 3–20 mmHg (or Torr) at 0 and 25 °C. Powder samples (about 80 mg) were loaded into a quartz sample tube and degassed by a sample degassing system (VacPrep 061 degasser, micromeritics, USA) at 150 °C under vacuum for 24 h before the sorption analysis. Then, the equilibrium weight of the powder sample was recorded and used in evaluating its CO_2 capture performance with the amount of CO_2 adsorbed at 850 Torr extracted from isotherms.

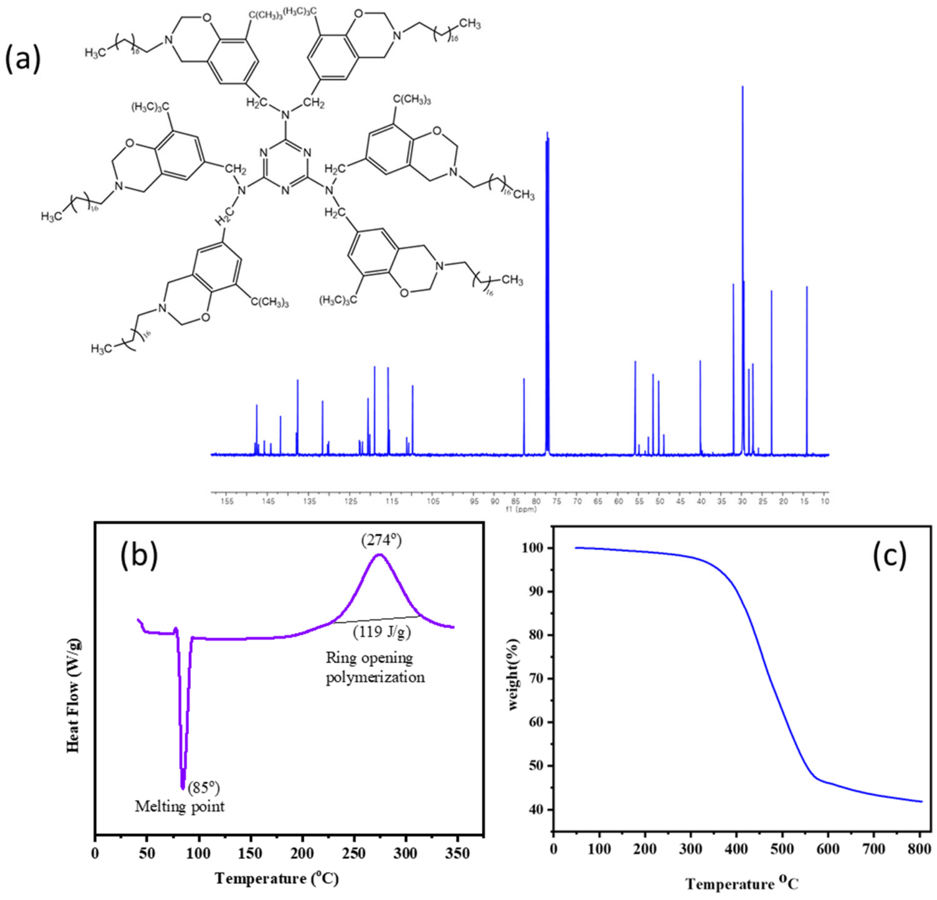

Figure S1. (**a**) ^{13}C-NMR (**b**) DSC of MSt-Bzo and (**c**), TGA of poly (MSt-Bzo).

Calculation of interplanar distance, d

Using Bragg's equation

$n\lambda = 2d\sin\theta$

Rearranging the above equation, we get

$d = n\lambda/2\sin\theta$

By substituting the values,

$n = 1$

$\lambda = 1.5406$ Å

$2\theta = 23.5°$ ($\theta = 11.75°$)

The interplanar distance, d, was found to be 0.378 nm
